# The emerging roles of autophagy in the homeostasis of lysosome-related organelles

**DOI:** 10.3389/fcell.2025.1638905

**Published:** 2025-09-26

**Authors:** Xinran Wang, Luan Zhang, Xueqin Cao, Yuting Zhao

**Affiliations:** ^1^ Institute of Future Agriculture, Northwest A&F University, Yangling, China; ^2^ College of Life Sciences, Northwest A&F University, Yangling, China

**Keywords:** melanophagy, lysosome-related organelle, autophagy, selective autophagy, selective autophagy receptor, non-canonical autophagy, secretory autophagy, LC3-associated phagocytosis

## Introduction

Canonical autophagy mediates the quality control of damaged organelles selectively, such as the clearance of mitochondria (mitophagy) and lysosomes (lysophagy) (Picca et al., 2023; Vargas et al., 2023). Selective autophagy receptors recognize organelle cargoes, some of which depend on cargo ubiquitination (Vargas et al., 2023). Lysosome-related organelles (LROs) are a variety of secretory compartments, including melanosomes in pigment cells, Weibel–Palade bodies (WPBs) in endothelial cells, lamellar bodies (LBs) in type II alveolar epithelial cells, major histocompatibility complex (MHC) class II compartments in antigen-presenting cells (APCs), and secretory granules (SGs) in mast cells (Delevoye et al., 2019). However, it remains unclear whether autophagy regulates the clearance of LROs or which autophagy receptors, if any, are involved. Two recent studies have uncovered the mechanisms underlying the selective autophagy of melanosomes (melanophagy)—the first studies on LRO selective autophagy (Lee et al., 2024; Park et al., 2024), which shares a common strategy with other types of selective autophagy. Noncanonical autophagy has been implicated in the formation, maturation, and secretion of various LROs (Ushio et al., 2011; Torisu et al., 2013; Ramkumar et al., 2017; Morishita et al., 2020; Li et al., 2022; Sarango et al., 2022; Omari et al., 2024), suggesting the complex roles of autophagy in LRO regulation, which requires in-depth research on LRO autophagy.

In this opinion piece, we compare the molecular mechanisms reported by recent studies on melanophagy. We also discuss the current understanding of the roles of autophagy, mostly noncanonical, in regulating LRO biogenesis and secretion, and we propose future studies investigating the role of autophagy in LRO homeostasis.

## Selective autophagy of melanosomes (melanophagy)

**FIGURE 1:**
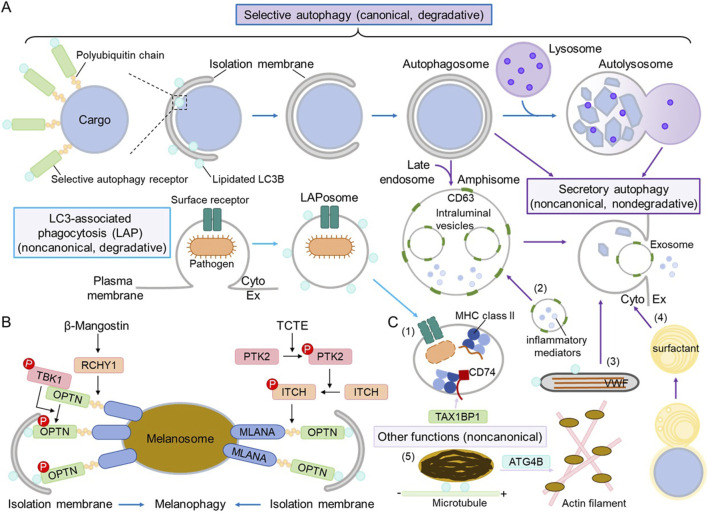
The roles of autophagy in maintaining homeostasis of lysosome-related organelles (LROs). **(A)** General scheme of selective autophagy, LC3-associated phagocytosis (LAP), and secretory autophagy. A selective autophagy receptor recognizes specific cargo substrates, which are usually polyubiquinated on cargo organelles, conferring the selectivity. The selective autophagy receptor recruits an isolation membrane by interacting with the lipidated ATG8/LC3/GABARAP family proteins, and the subsequent events (isolation membrane growth and closure, autophagosome formation and fusion with a lysosome, and autolysosome formation and degradation; dark blue arrows) are the same as those of canonical, degradative autophagy. During LAP, the surface receptor recognizes an extracellular pathogen, and then a LAPosome decorated with lipidated LC3 is formed to internalize pathogens before fusing with a lysosome and degrading the cargo (light blue arrows). Secretory autophagy consists of various processes, where autophagosomes, amphisomes (autophagosomes that have fused with late endosomes), or autolysosomes may fuse with the plasma membrane to release their contents (dark purple arrows). “Cyto” refers to the cytoplasm, whereas Ex refers to the extracellular space. For simplicity, the lipidated LC3 is not shown in autophagosomes, amphisomes, or autolysosomes. **(B)** Mechanisms of melanophagy. Two recent studies revealed the molecular mechanisms of selective autophagy of melanosomes—melanophagy. Upon β-mangostin stress, melanosome proteins are polyubiquinated by the E3 ubiquitin ligase RCHY1 and recognized by the selective autophagy receptor OPTN, which recruits the active protein kinase TBK1 and gets phosphorylated. Upon TCTE stress, melanosome protein MLANA is polyubiquinated by the E3 ubiquitin ligase ITCH, which gets phosphorylated by the active protein kinase PTK2, and it is also recognized by OPTN. OPTN interacts with lipidated LC3B on the isolation membrane to target melanosomes for degradation via canonical autophagy. **(C)** Different roles of autophagy in maintaining LRO homeostasis. (1) LAP regulates the loading of antigen peptides into MHC class II compartments in antigen-presenting cells. TAX1BP1 plays a noncanonical role in stabilizing CD74/MHC class II for proper presentation (light purple arrow). (2) In mast cells, CD63-positive secretory granules fuse with amphisomes and release inflammatory mediators and exosomes. (3) In endothelial cells, secretory autophagy regulates the release of the von Willebrand factor (VWF) from Weibel–Palade bodies. (4) In type II alveolar epithelial cells, fusion with autophagosomes leads to the maturation of lamellar bodies, and secretory autophagy regulates the release of surfactant. (5) In melanocytes, LC3B and ATG4B regulate melanosome transport on microtubules and actin filaments, respectively; this process is considered noncanonical as autophagic degradation is not involved (light purple arrow).

Selective autophagy of damaged organelles has been extensively studied (Vargas et al., 2023). In general, after cellular organelles get damaged by stress, specific E3 ligases mediate the polyubiquitination of specific cargo substrates on or in the organelles which become accessible upon damage; selective autophagy receptors interact with both polyubiquitinated cargo substrates and lipidated ATG8/LC3/GABARAP family proteins to recruit an isolation membrane to the cargo—the damaged organelles; with isolation membrane growth and closure, an autophagosome enclosing the cargo is formed, which then fuses with a lysosome, leading to the degradation of the cargo inside (Figure 1A). This process shares the same machinery as bulk or nonselective autophagy, including the ULK1 complex, ATG9, the class III phosphatidylinositol 3-kinase (PI3K) complex, and the ATG8 conjugation system. However, some selective autophagy receptors are resident proteins in the cargo organelles (i.e., ER-phagy); thus, polyubiquitination is not required (Vargas et al., 2023). Phosphorylation of E3 ubiquitin ligases or selective autophagy receptors by certain protein kinases can further regulate the process of selective autophagy (Vargas et al., 2023).

It is surprising that little is known about whether selective autophagy of LROs occurs or not. Melanosomes are LROs found in pigment cells, such as skin melanocytes and retinal pigment epithelial cells. Recently, two groups of researchers reported that autophagy plays a canonical role in melanosome degradation (Park et al., 2020; Lee et al., 2024; Park et al., 2024), demonstrating the first example of LRO selective autophagy. β-Mangostin reduces the amount of intracellular and extracellular f melanosomes in α-melanocyte-stimulating hormone (MSH)-stimulated melanocytes, and this effect is reversed by autophagy inhibition via ATG5 (a component of the ATG8-conjugation system) knockdown, FIP200 (a subunit of the ULK1 complex) knockdown, or 3-methyladenine treatment (a class III PI3K inhibitor) (Lee et al., 2024). Similarly, 3,4,5-trimethoxycinnamate thymol ester (TCTE) inhibits skin pigmentation in an autophagy-dependent manner, as TCTE reduces α-MSH-stimulated pigmentation, and this reduction is restored by ATG5 knockdown (Park et al., 2020). Furthermore, β-mangostin induces the degradation of melanosomes but not of mitochondria, the endoplasmic reticulum (ER), or peroxisomes, indicating that the process is selective (Lee et al., 2024). If β-mangostin and TCTE induce selective autophagy of melanosomes (melanophagy), which selective autophagy receptors and possible E3 ubiquitin ligases participate in the process?

As summarized in Table 1, OPTN/optineurin has been identified as the melanophagy receptor. It was screened alongside several known selective autophagy receptors, including NBR1, SQSTM1/p62, FUNDC1, NDP52, NIX, and TAX1BP1 (Lee et al., 2024; Park et al., 2024). K63-linked polyubiquitination of total melanosome proteins increases with β-mangostin treatment, while polyubiquitination of the melanosome marker MLANA/Melan-A increases with TCTE stimulation. OPTN co-localizes with melanosomes via its ubiquitin-binding domain (UBD) and interacts with MLANA in response to stress, completing the step of cargo recognition. OPTN also binds to lipidated LC3B to recruit the isolation membrane to melanosomes. The E3 ubiquitin ligases RCHY1 and ITCH have been identified to regulate the polyubiquitination of melanosome cargo substrates. TBK1 phosphorylates OPTN (on mouse Ser 187, corresponding to human Ser 177) to activate OPTN during β-mangostin-induced melanophagy, and OPTN is also required for TBK1 activation on melanosomes. PTK2 phosphorylates ITCH to promote the polyubiquitination of MLANA during TCTE-induced melanophagy. Taken together, the molecular mechanism of melanophagy follows the selective autophagy paradigm, with two pathways being characterized: β-mangostin–RCHY1–TBK1–OPTN and TCTE–PTK2–ITCH–MLANA–OPTN (Figure 1B).

**TABLE 1:** Comparison of two recent studies on melanophagy.

Research	Lee et al. (2024)	Park et al. (2024)
Stress	β-Mangostin	3,4,5-Trimethoxycinnamate thymol ester (TCTE)
Cell type	Melanocyte (B16F10)	Melanocyte (B16F10)
Cargo for degradation	Melanosome	Melanosome
Selective autophagy receptor	OPTN	OPTN
ATG8 protein	LC3B	LC3B
Polyubiquitinated cargo substrate	K63-linked, substrates not specified	MLANA
E3 ubiquitin ligase	RCHY1	ITCH
Upstream kinase	TBK1	PTK2
Phosphorylated substrate (site)	OPTN (mouse S187)	ITCH (site not specified)
Inhibitor	ATG5↓, FIP200↓ 3-Methyladenine (→class III PI3K) BX-795 (→TBK1)	ATG5↓ Dichlone (→ITCH) Y15 (→PTK2)

It is uncertain whether selective autophagy of other types of LROs exists or not. Since LROs are usually too big for efficient proteasomal degradation, we think it is highly likely that autophagic/lysosomal degradation is utilized to clear unwanted or damaged LROs. Future studies can utilize similar strategies as in the abovementioned melanophagy studies to identify the selective autophagy receptors and regulators of other LROs.

## The roles of noncanonical autophagy in regulating the biogenesis and secretion of LROs

The canonical role of autophagy is to recognize cargoes, pack them in double-membraned autophagosomes, and target them for lysosomal degradation, which is crucial for quality control of organelles under stress, as in the processes of melanophagy. During the past decade, the noncanonical (non-degradative or autophagosome-independent) role of autophagy has been extensively investigated (Piletic et al., 2023; Deretic et al., 2024) and implicated in regulating the internalization of extracellular components, the secretion of soluble or membrane-enclosed cargoes, and the noncanonical functions of autophagy proteins. Previous studies have suggested that autophagy plays distinct roles in the biogenesis, maturation, motility, and secretion of LROs, the majority of which are noncanonical (Figure 1C).

During the biogenesis of melanosomes (melanogenesis), UVRAG, a subunit of the class III PI3K complex 2 that mediates autophagosome maturation into autolysosomes, is required for cell pigmentation independent of class III PI3K complex 2 activity; instead, UVRAG interacts with the BLOC-1 complex and regulates the stability and distribution of BLOC-1, leading to proper melanogenic cargo-sorting (Yang et al., 2018). LC3B, a major ATG8 family protein in autophagosomes, localizes to melanosomes to facilitate transport on microtubules, whereas ATG4B, which regulates LC3B lipidation and delipidation, mediates melanosome translocation to actin filaments and transport (Ramkumar et al., 2017). Beclin 1, another subunit of the class III PI3K complex, along with LC3B and ATG7, has been suggested to activate MITF, the major transcription factor for melanogenic gene expression; however, the underlying molecular mechanisms remain unknown (Lee et al., 2022).

MHC class II molecules, which are mainly expressed in B cells, monocytes, macrophages, and dendritic cells, among others, present antigenic peptides on the cell surface to CD4^+^ T cells (Rock et al., 2016; Pishesha et al., 2022). The antigenic peptides presented by MHC class II molecules are processed in specialized endolysosomal compartments, MHC class II compartments, and they are loaded onto MHC class II molecules for presentation. These processes involve both canonical and noncanonical autophagy (Münz, 2022). The autophagy receptor TAX1BP1 not only facilitates autophagic degradation of intracellular antigens and their delivery to MHC class II compartments but also stabilizes the invariant chain CD74/MHC class II complex to ensure the proper presentation of high-affinity peptides (Sarango et al., 2022). LC3-associated phagocytosis (LAP) is a type of noncanonical autophagy (degradative, autophagosome-independent) and functions in immune responses, where ATG8/LC3 is conjugated to single-membraned phagosomes, relying on a subset of canonical autophagy machinery such as the UVRAG/Beclin 1-containing class III PI3K complex and the ATG8-conjugation system but not the ULK1 complex (Peña-Martinez et al., 2022). LAP accelerates extracellular antigen internalization and processing for MHC class II under the regulation of ATG4B oxidation (Ligeon et al., 2021; Münz, 2022).

Secretory autophagy or autophagy-dependent secretion (New and Thomas, 2019; Piletic et al., 2023), another type of noncanonical autophagy (non-degradative, most likely autophagosome-dependent), regulates the content release of several LROs, including WPBs in endothelial cells, LBs in type II alveolar epithelial cells, and SGs in mast cells. The secretion of the von Willebrand factor (VWF) from WPBs requires the autophagy machinery because autophagy inhibition (through ATG5 or ATG7 knockdown, or by using inhibitors of lysosomes/autolysosomes, such as chloroquine or bafilomycin A1) blocks VWF secretion. WPBs are also found close to or within LC3-positive autophagosomes (Torisu et al., 2013). Similarly, the secretion of surfactant from lung LBs relies on autophagy because autophagy inhibition (through FIP200 or ATG7 knockout, or by using 3-Methyladenine treatment) impairs lung LB maturation and surfactant protein secretion. Furthermore, lung LBs fuse with LC3B-positive autophagosomes (Morishita et al., 2020; Li et al., 2022). The degranulation process of mast cells, during which mast cells release inflammatory mediators such as histamine and β-hexosaminidase from SGs upon antigen stimulation, is also autophagy (ATG7)-dependent (Ushio et al., 2011). CD63-positive SGs fuse with LC3-positive late endosomes (amphisomes) and release exosomes upon stimulation (Omari et al., 2024).

## Investigating the roles of autophagy in regulating the homeostasis of LROs

As discussed above, autophagy and autophagy proteins participate in different aspects of LRO homeostasis, ranging from the formation and cargo sorting of LROs to the content release and breakdown of LROs (Figure 1). We believe that crucial questions should be addressed in the field, such as whether selective autophagy of LROs other than melanosomes takes place, what the molecular mechanisms underlying noncanonical autophagy of LROs are, and how canonical and noncanonical autophagy are coordinated in the same type of LROs.

In addition to screening known autophagy receptors, as in melanophagy studies, we propose that LRO cargoes and autophagy receptors be investigated using a proteomic approach. In autophagy-deficient cells (e.g., with FIP200 or ATG5 deletion), cargoes and autophagy receptors shall decrease in lysosomes and increase in whole cells. Therefore, proteomic analyses of purified lysosomes (i.e., through LysoIP (Abu-Remaileh et al., 2017)) and of the whole-cell lysate from autophagy-sufficient and autophagy-deficient cells can help to narrow down the list of cargoes and autophagy receptors (Herhaus et al., 2024). Candidate LRO cargoes and autophagy receptors can then be found by comparing them with LRO proteomes. Alternatively, ATG8 family proteins can be used as bait to search for autophagy receptors in purified LROs. To validate whether a candidate autophagy receptor is selective for certain LROs, the following criteria should be met: (1) deletion of the candidate autophagy receptor increases the level of LRO cargoes, (2) the candidate autophagy receptor localizes to the LROs, and this co-localization is likely enhanced under stress, (3) the candidate autophagy receptor interacts with ATG8 family proteins, and (4) the candidate autophagy receptor is a resident protein of the LROs, or interacts with LRO cargoes in a polyubiquitination-dependent manner. Regarding the MHC class II compartments, known lysophagy receptors (TAX1BP1 and p62) should be on the shortlist.

The autophagy machinery can regulate the internalization of extracellular components during LC3-associated phagocytosis (LAP), micropinocytosis (LAM), and endocytosis (LANDO) processes (Magné and Green, 2022; Deretic et al., 2024). It would be interesting to test whether LAM or LANDO impacts MHC class II compartments in addition to LAP. Secretory autophagy also consists of different processes, such as LC3-dependent extracellular vesicle loading and secretion (LDELS) and secretory autophagy during lysosome inhibition (SALI), among others (Debnath and Leidal, 2022; Deretic et al., 2024). It is important to elucidate the molecular details of the secretory autophagy in WPBs, lung LBs, and mast cell SGs. These LROs co-localize with LC3 and require ATG7 (a component of the ATG8-conjugation system) for secretion; however, little is known about whether upstream autophagy proteins such as the ULK1 complex, ATG9, or the class III PI3K complex are necessary for secretion and whether induction of autophagy is sufficient to promote the secretion of these LROs. Damaged mitochondria can be released into the extracellular space in an autophagy-dependent manner rather than degradation via mitophagy (Nicolás-Avila et al., 2020; Gong et al., 2024). It will be intriguing if damaged LROs could be released from cells, similar to damaged mitochondria, serving as an alternative path for clearance.

With a better understanding of how canonical and non-canonical autophagy regulate different stages of the life cycle of LROs, we expect that key autophagy proteins and modulators that coordinate the different roles of autophagy in maintaining the homeostasis of LROs will soon be uncovered.
